# From Type 2 Diabetes Mellitus to Autoimmune Failure: Euglycemic Diabetic Ketoacidosis Revealing Latent Autoimmune Diabetes in Adults

**DOI:** 10.7759/cureus.105361

**Published:** 2026-03-17

**Authors:** Venkata Yashashwini Maram Reddy, Venkata Sai rithin Maramreddy, Reddy Jhansi Ramireddy

**Affiliations:** 1 Internal Medicine, Guntur Medical College, Guntur, IND; 2 Medicine, Narayana Medical College, Nellore, IND; 3 Pediatrics, Kurnool Medical Colllege, Kurnool, IND

**Keywords:** autoimmune diabetes, diabetes mellitus type 1.5, euglycemic diabetic ketoacidosis, euglycemic dka, insulin, ketone-prone diabetes, latent autoimmune diabetes in adults (lada), sodium-glucose cotransporter-2 (sglt2) inhibitors, type 1 diabetes mellitus, type 2 diabetes mellitus

## Abstract

Latent autoimmune diabetes in adults (LADA) is a slowly progressive form of autoimmune diabetes that is frequently misclassified as type 2 diabetes mellitus (T2DM) because of its gradual onset and initial responsiveness to oral hypoglycemic agents. This misclassification may delay appropriate insulin therapy and expose patients to medications that increase the risk of metabolic complications. Sodium glucose cotransporter-2 (SGLT2) inhibitors have been increasingly associated with euglycemic diabetic ketoacidosis (DKA), particularly in individuals with underlying insulin deficiency. Several reports describe euglycemic DKA as the presenting manifestation of previously unrecognized LADA.

We report a case of a 55-year-old man with a nine-year history of presumed T2DM treated with metformin and canagliflozin who presented with vomiting, dehydration, and metabolic acidosis. Laboratory evaluation revealed high anion gap metabolic acidosis and marked ketosis despite only modest hyperglycemia, consistent with euglycemic DKA. The patient was successfully treated with intravenous fluids, insulin infusion, and electrolyte replacement. Further evaluation demonstrated markedly elevated anti-glutamic acid decarboxylase (GAD65) antibodies and reduced C-peptide levels, confirming autoimmune β-cell failure consistent with LADA. The SGLT2 inhibitor was discontinued, and the patient was transitioned to a basal-bolus insulin regimen with structured diabetes education.

This case highlights how slowly progressive autoimmune diabetes may remain unrecognized for years and may be unmasked by metabolic stressors or SGLT2 inhibitor therapy. Clinicians should consider autoimmune diabetes in adults with atypical diabetes phenotypes or unexpected ketoacidosis and use antibody testing and C-peptide measurement to guide diagnosis and management.

## Introduction

Diabetes mellitus (DM) is increasingly recognized as a heterogeneous metabolic disorder with overlapping clinical phenotypes that may lead to misclassification between autoimmune and non-autoimmune forms [1]. Latent autoimmune diabetes in adults (LADA) represents the most common form of autoimmune diabetes in adulthood and is frequently misdiagnosed as type 2 DM (T2DM) due to its insidious onset and initial insulin independence [2,3].

LADA is defined by adult-onset diabetes (typically >30 years of age), the presence of at least one pancreatic islet autoantibody, most commonly glutamic acid decarboxylase antibodies (GADA), and the absence of insulin requirement for at least six months after diagnosis (Immunology of Diabetes Society criteria) [2]. Epidemiological studies estimate that LADA accounts for approximately 5%-12% of patients initially diagnosed with T2DM, making it the second most common form of diabetes after T2DM and the most prevalent autoimmune diabetes in adults [2,4]. Despite this, routine antibody screening is not universally performed, contributing to delayed diagnosis and suboptimal management [5].

Pathophysiologically, LADA shares genetic and immunologic features with type 1 DM (T1DM), including human leukocyte antigen (HLA) susceptibility and progressive autoimmune β-cell destruction, but with a slower rate of decline in endogenous insulin secretion [6]. This gradual loss of β-cell function explains the initial response to oral hypoglycemic agents followed by eventual insulin dependence [7].

Misclassification has important therapeutic implications. Sodium glucose cotransporter-2 (SGLT2) inhibitors, while effective in T2DM, have been associated with an increased risk of diabetic ketoacidosis (DKA), including euglycemic DKA, particularly in patients with underlying insulin deficiency [8]. Euglycemic DKA is characterized by high anion gap metabolic acidosis and ketosis with relatively normal or mildly elevated blood glucose levels (<250 mg/dL), which may delay recognition and treatment. In patients with unrecognized LADA, SGLT2 inhibitor-induced reductions in circulating insulin and enhanced glucagon-mediated ketogenesis may precipitate this complication [2,8].

We report a case of euglycemic DKA in a 55-year-old man with a nine-year history of presumed T2DM treated with metformin and canagliflozin, ultimately found to have autoimmune insulin deficiency consistent with LADA. This case underscores the importance of early antibody testing in adults with atypical features of T2DM and highlights the metabolic risks associated with SGLT2 inhibitor therapy in patients with unrecognized autoimmune diabetes.

## Case presentation

A 55-year-old male patient presented to the emergency department with a one-day history of vomiting, generalized weakness, polyuria, and polydipsia. He had a nine-year history of presumed T2DM treated with metformin 500 mg twice daily and canagliflozin 100 mg daily as part of his long-term outpatient regimen, along with stage 3 diabetic nephropathy (estimated glomerular filtration rate (eGFR) 41 mL/min/1.73 m²). He also had long-standing hypertension treated with telmisartan 40 mg daily and gastroesophageal reflux disease managed with pantoprazole 40 mg daily. Family history was notable for hypertension in his father, T2DM in his mother, and Hashimoto's thyroiditis in his sister. The patient reported light alcohol use (one to two drinks per week) and minimal tobacco exposure (<1 pack year). There was no history of illicit drug use. He denied fever, symptoms suggestive of infection, recent medication noncompliance, steroid use, chest pain, shortness of breath, or prior episodes of diabetic ketoacidosis.

On examination, the patient appeared dehydrated and ill. He was alert but confused about time, place, person, and situation. Vital signs revealed a heart rate of 112 beats per minute (bpm), blood pressure of 96/60 mmHg, respiratory rate of 24 breaths per minute with deep breathing, oxygen saturation of 98% on room air, and temperature of 36.8°C. The calculated body mass index was 24 kg/m². Oral mucosa was dry with reduced skin turgor, and a noticeable ketone breath odor was present. Abdominal examination revealed mild epigastric tenderness without guarding or rigidity. Cardiovascular and respiratory examinations were otherwise unremarkable.

Laboratory studies upon admission are shown in Table 1. 

**Table 1:** The patient's laboratory findings were consistent with euglycemic diabetic ketoacidosis, including high anion gap metabolic acidosis, ketosis, and mild renal and electrolyte abnormalities.

Investigation	Result	Reference Range
Random blood glucose	186 mg per dL	70 to 140 mg per dL
HbA1c	7.2%	less than 5.7%
Serum sodium	130 mmol per L	135 to 145 mmol per L
Serum potassium	5.6 mmol per L	3.5 to 5 mmol per L
Serum chlorine	96 mmol per L	98 to 106 mmol per L
Anion gap	24 mmol per L	8 to 16 mmol per L
Serum ketones beta-hydroxybutyrate	4.8 mmol per L	less than 0.6 mmol per L
Serum osmolality	295 mOsm per kg	275 to 295 mOsm per kg
Blood urea nitrogen	42 mg per dL	7 to 20 mg per dL
Serum creatinine	1.9 mg per dL	0.6 to 1.3 mg per dL
Serum lactate	1.6 mmol per L	0.5 to 2.2 mmol per L
Arterial blood gas		
Arterial pH	7.18	7.35 to 7.45
Partial pressure of carbon dioxide	24 mmHg	35 to 45 mmHg
Partial pressure of oxygen	98 mmHg	80 to 100 mmHg
Serum bicarbonate	10 mmol per L	8 to 16 mmol per L
Base excess	-14	-2 to +2
Urine analysis		
Urine ketones	3 plus	Negative
Urine glucose	2 plus	Negative
Urine protein	1 plus	Negative
Urine specific gravity	1.030	1.005 to 1.030
Urine microscopy	No active sediment	None
Leucocytes	Negative	Negative
Nitrates	Negative	Negative

After confirmation of DKA in the emergency department, the patient was started on intravenous 0.45% normal saline with 5% dextrose, intravenous regular insulin infusion, and potassium supplementation. He was shifted to the intensive care unit for close monitoring. Canagliflozin and metformin were discontinued, and blood glucose was monitored continuously. The anion gap closed with normalization of acid-base status. Over three days, the patient showed marked clinical improvement with resolution of ketosis and normalization of serum lactate and osmolality. He was transitioned to a subcutaneous basal bolus insulin regimen and subsequently transferred to the inpatient unit in stable condition.

In view of relatively preserved blood glucose levels in DKA, additional investigations were performed to assess endogenous insulin secretion, evaluate for autoimmune diabetes, and exclude alternative causes of ketosis, as shown in Table 2.

**Table 2:** This values in this table confirm autoimmune insulin deficiency and exclude toxic or secondary causes of ketoacidosis.

Investigation	Result	Reference Range
Fasting insulin level	1.1 microIU per mL	2 to 25 microIU per mL
Serum C peptide	0.6 ng per mL	0.9 to 7.1 ng per mL
Anti GAD65 antibodies	99 U/mL	Less than 5 U/mL
Urine drug screen	Negative	Negative
Serum toxicology screen	Negative	Negative
Thyroid stimulating hormone	3.4 microIU per mL	0.4 to 4.5 microIU per mL
Morning serum cortisol	16 microg per dL	6 to 23 microg per dL

Following resolution of euglycemic DKA, long-term therapy was restructured to reflect confirmed LADA. The SGLT2 inhibitor was permanently discontinued given the established risk of recurrent ketoacidosis in insulin-deficient states. The patient was transitioned to a basal bolus insulin regimen with dose titration guided by capillary glucose monitoring.

Metformin was cautiously reintroduced in the setting of preserved renal function to reduce insulin requirements. Structured education was provided regarding carbohydrate counting, hypoglycemia recognition, sick-day management, and home ketone monitoring, given prior susceptibility to ketosis.

Given stage 3 diabetic nephropathy and the recognized cardiovascular risk associated with LADA, cardiovascular risk reduction strategies were intensified, including optimization of antihypertensive therapy and statin treatment. Ongoing follow-up was arranged at three-month intervals to monitor glycemic control, insulin requirements, renal function, and potential progression of autoimmune β-cell failure. Screening for associated autoimmune conditions, particularly thyroid disease, was recommended for outpatient follow-up.

## Discussion

Epidemiology and clinical importance of LADA

LADA is the most common form of autoimmune diabetes in adults and accounts for approximately 5%-12% of individuals initially classified as having T2DM [2-4]. Increasing recognition of diabetes heterogeneity has challenged the traditional classification of T1DM and T2DM, particularly in adults presenting without classic phenotypic features [1].

Despite its prevalence, LADA remains substantially underdiagnosed because routine pancreatic autoantibody screening is not universally recommended in adult-onset diabetes [5]. As a result, many patients are managed as T2DM for years before progressive β-cell failure becomes clinically evident. Misclassification carries therapeutic implications, including inappropriate reliance on oral hypoglycemic agents and exposure to medications that may increase metabolic risk in insulin-deficient states [2].

Diagnostic criteria and immunopathogenesis: why β-cell failure progresses slowly

According to the Immunology of Diabetes Society, LADA is defined by adult-onset diabetes, the presence of at least one islet autoantibody, and the absence of an insulin requirement for at least six months after diagnosis [2]. GAD65 antibodies are the most sensitive marker in adult autoimmune diabetes and are central to risk stratification [3]. Epitope specificity and HLA-DR-DQ genotype further refine prognostic assessment and predict β-cell decline [9].

LADA demonstrates substantial heterogeneity. Individuals with high GAD titers exhibit more rapid β-cell loss and immunologic resemblance to T1DM, whereas low-titer patients display more metabolic features overlapping with T2DM [3]. Genetic risk scoring further supports that LADA is genetically closer to T1DM than T2DM, although the total T1DM risk allele burden is lower than in childhood-onset T1DM, potentially explaining its later onset [9]. 

LADA shares key immunologic features with T1DM but progresses more gradually. Dysregulated T-cell phenotypes reflecting impaired peripheral immune regulation have been described in LADA, suggesting a slower but persistent autoimmune process [10]. B lymphocytes also contribute to loss of self-tolerance and β-cell destruction, with altered regulatory B-cell populations demonstrating a mixed immunologic profile overlapping T1DM and T2DM [11].

Emerging mechanistic models conceptualize autoimmune diabetes as an inflammatory inhibitory set-point continuum involving NF-κB/EGFR-JAK/STAT signaling gradients and HLA-KIR interactions, which may explain differences in aggressiveness between childhood-onset T1DM and adult-onset LADA [12]. Additionally, autoantibodies against interferon-α have been proposed as potential modulators of autoimmune intensity in LADA compared with classic T1DM [13].

Genetic and metabolic interactions: why it looks like T2DM leading to diagnostic delay

LADA reflects a genetic admixture of autoimmune and metabolic susceptibility loci. Interaction between HLA genotypes and metabolic factors, such as overweight status, may produce hybrid autoimmune and metabolic phenotypes that resemble T2DM clinically [14]. Furthermore, epidemiologic data suggest that early-life factors such as low birthweight combined with adult overweight status may increase LADA risk [15], reinforcing the concept that metabolic stress can accelerate autoimmune expression.

This interaction likely contributed to diagnostic delay in our patient, whose adult age at onset, absence of initial ketoacidosis, and intermediate body mass index obscured underlying autoimmunity. Routine antibody screening is not standard in adult-onset diabetes, further perpetuating misclassification [5].

The overlapping clinical and metabolic characteristics of T1DM, T2DM, and LADA contribute significantly to diagnostic uncertainty. A structured comparison of immunogenetic, immunophenotypic, metabolic, and prognostic features is summarized in Table 3 to clarify these distinctions and their therapeutic implications.

**Table 3:** This table summarizes the key clinical, immunologic, genetic, and therapeutic distinctions between T1DM, T2DM, and LADA. Abbreviations: CV: cardiovascular; CVD, cardiovascular disease; DKA, diabetic ketoacidosis; GAD, glutamic acid decarboxylase; HLA, human leukocyte antigen; IFN, interferon; LADA, latent autoimmune diabetes in adults; T1DM, type 1 diabetes mellitus; T2DM, type 2 diabetes mellitus.

Domain	Type 1 Diabetes (T1DM)	Type 2 Diabetes (T2DM)	LADA
Typical onset pattern [1-4]	Often acute, DKA can be a presenting feature	Gradual and often incidentally discovered	Gradual
Core pathobiology [2,7]	Autoimmune β-cell destruction and absolute insulin deficiency	β-cell dysfunction and Insulin resistance	Autoimmune β-cell destruction (slower) and variable insulin resistance
Islet autoantibodies [2,3]	Usually present (GAD, IA-2, ZnT8, IAA)	Absent	Present; GAD65 is the most common
C-peptide trajectory [2,7]	Low/absent early, rapidly declines	Normal/high early, declines later	Low normal initially, progressive decline
Genetic architecture [6]	Strong autoimmune genetic risk (HLA class II prominent)	Polygenic metabolic risk	Closer to T1DM than T2DM genetically
Immune phenotyping (T cells) [10]	Strong pro-inflammatory autoimmune signatures with cytotoxic T cell involvement	No consistent islet-directed autoimmunity	Altered regulatory/effector T-cell phenotypes
Immune phenotyping (B cells) [11]	Autoantibody production	No islet cell autoimmunity	Altered peripheral B-cell subsets support a mixed autoimmune profile
Interferon-related markers [12]	Early-onset T1DM may show a more aggressive immune phenotype	Not typical	IFN-α neutralizing antibodies may distinguish LADA from early-onset T1DM (supports “less aggressive autoimmunity” concept)
Clinical phenotype early course [2-4]	Leaner on average	Overweight/obesity common	Variable BMI; may look T2DM-like initially
Acute ketoacidosis risk [2,8]	High if insulin is interrupted	Lower	Risk increases as insulin deficiency progresses
Long-term prognosis (vascular outcomes) [16,17]	High microvascular risk with poor control, CVD risk is significant	High CVD and microvascular risk	Equal or higher risks of death, CVD, and microvascular disease
Treatment foundation [2]	Insulin from diagnosis	Lifestyle and non-insulin agents, insulin later if needed	Treat as progressive insulin deficiency, early insulin often needed and tailored by C-peptide trajectory
Therapy cautions with SGLT2 inhibitors [2,8]	SGLT2 is sometimes used with caution in T1DM	SGLT2 is often beneficial in T2DM (CV/renal)	Heightened caution with SGLT2 due to DKA risk in the insulin-deficient phenotype
Emerging disease-modifying direction [9,12,13]	Research focusing on Immunomodulation	Metabolic, weight-loss, and insulin sensitization	Risk stratification using immune/genetic markers and immunophenotype-informed precision approaches is being explored

As shown in Table 3, LADA aligns immunogenetically with T1DM yet frequently presents with metabolic characteristics overlapping with T2DM. This duality explains both diagnostic delay and therapeutic vulnerability.

Mechanistic basis of SGLT2 inhibitor-associated euglycemic DKA

In patients with partial insulin deficiency, SGLT2 inhibitors can precipitate ketoacidosis through combined metabolic effects. By promoting urinary glucose excretion, these agents reduce plasma glucose but also lower circulating insulin levels and increase glucagon secretion. The altered insulin-to-glucagon ratio enhances lipolysis, increases hepatic free fatty acid flux, and stimulates ketogenesis. Because glycosuria limits hyperglycemia, significant ketosis may develop despite blood glucose levels remaining below 250 mg/dL, resulting in euglycemic DKA [8].

In the setting of progressive autoimmune β-cell failure, as in this patient, SGLT2 inhibition may unmask previously compensated insulin deficiency. This interaction underscores the importance of evaluating endogenous insulin reserve before or during SGLT2 inhibitor therapy in atypical diabetes phenotypes [8].

Long-term complications and prognostic implications

Contrary to earlier assumptions that LADA may be metabolically milder than T2DM, emerging data indicate comparable or higher cardiovascular and mortality risks. Population-based analyses demonstrate equal to increased risks of death, cardiovascular disease, and retinopathy in LADA compared with T2DM, particularly in high GAD titer subgroups [16]. Additionally, individuals with LADA show a higher prevalence of dyslipidemia, hypertension, diabetic kidney disease, and peripheral neuropathy [17].

Current treatment and guideline-based management

Management of LADA should be aligned with progressive insulin deficiency rather than insulin resistance alone. The international expert panel consensus emphasizes individualized therapy based on C-peptide levels, metabolic phenotype, and rate of β-cell decline [2]. In patients with reduced or declining C-peptide, such as in this case, early transition to insulin-based therapy is appropriate to minimize glucotoxicity and prevent ketosis [1,2].

Adjunctive therapies may be considered selectively. The combination of insulin with DPP-4 inhibitors has shown potential to improve glycemic control and preserve β-cell function compared with insulin alone in LADA cohorts, although evidence remains limited and heterogeneous [2]. GLP-1 receptor agonists have also been explored in LADA and may improve glycemic control in patients with residual β-cell function, although evidence remains limited [2]. Emerging immunomodulatory strategies, including intralymphatic GAD-alum therapy, have demonstrated safety and immunologic impact with preservation of β-cell function in genetically susceptible subgroups, suggesting a possible future disease-modifying role [14,15]. However, these approaches remain investigational.

Critically, this case reinforces the need for caution with SGLT2 inhibitors in patients with suspected insulin deficiency. Assessment of endogenous insulin reserve and autoimmune status may be prudent in atypical adult-onset diabetes before initiation of agents associated with DKA risk [2,8]. Additionally, metformin was continued to be given in preserved renal function to improve insulin sensitivity and reduce insulin requirements, although it does not modify autoimmune progression in LADA [2].

Prognosis and long-term follow-up

Prognosis in LADA depends on autoimmune intensity, residual β-cell function, and adequacy of metabolic control. Autoantibody burden and HLA background may influence the rate of progression to insulin dependence [9]. Long-term management should therefore include periodic reassessment of glycemic control, insulin requirements, and cardiovascular risk factors. Given the association of LADA with autoimmune clustering, clinicians should maintain vigilance for coexisting autoimmune conditions, particularly thyroid disease, guided by clinical context [2].

In the present case, the coexistence of stage 3 diabetic nephropathy and an episode of euglycemic DKA identifies a high-risk phenotype requiring structured education, insulin optimization, and aggressive cardiovascular risk reduction. Earlier recognition of autoimmune diabetes might have altered therapeutic decisions and potentially reduced metabolic instability.

## Conclusions

This case highlights the diagnostic challenges of LADA and the potential consequences of prolonged misclassification as T2DM. Slowly progressive autoimmune β-cell failure may remain unrecognized for years, delaying appropriate insulin therapy and exposing patients to medications that may increase metabolic risk. In this patient, treatment with an SGLT2 inhibitor likely contributed to the development of euglycemic DKA in the setting of underlying insulin deficiency. Clinicians should consider pancreatic autoantibody testing in adults with atypical diabetes phenotypes or unexpected metabolic complications, while the measurement of C-peptide can help assess endogenous insulin reserve and guide treatment decisions. Greater awareness of LADA and the development of practical screening strategies for adult-onset diabetes may help reduce diagnostic delay. Early recognition also allows appropriate medication selection, patient education on ketone monitoring, and structured long-term follow-up to optimize metabolic control and reduce complications. Future studies should focus on developing practical screening strategies for identifying LADA in adults with newly diagnosed diabetes, particularly before initiating therapies such as SGLT2 inhibitors that may increase the risk of ketoacidosis.
